# Gas in scattering media absorption spectroscopy as a potential tool in neonatal respiratory care

**DOI:** 10.1038/s41390-022-02110-y

**Published:** 2022-05-23

**Authors:** Jurate Panaviene, Andrea Pacheco, Christoph E. Schwarz, Konstantin Grygoryev, Stefan Andersson-Engels, Eugene M. Dempsey

**Affiliations:** 1 INFANT Research Centre, University College Cork, Cork, Ireland; 2 Department of Neonatology, Cork University Maternity Hospital, Cork, Ireland; 3 Department of Paediatrics and Child Health, University College Cork, Cork, Ireland; 4 Biophotonics@Tyndall, Irish Photonic Integration Centre, Tyndall National Institute, University College Cork Lee Maltings, Dyke Parade, Cork, Ireland; 5 Department of Physics, University College Cork, Cork, Ireland; 6 Department of Neonatology, University Children’s Hospital, Tübingen, Germany

## Abstract

**Abstract:**

Gas in scattering media absorption spectroscopy (GASMAS) is a novel optical technology employing near-infrared light. It has a potential use in the medical setting as a monitoring and diagnostic tool by detecting molecular oxygen within gas pockets and thus may be a useful adjunct in respiratory monitoring. GASMAS has potential advantages over other monitoring devices currently used in clinical practice. It is a non-invasive, continuous, non-ionising technology and provides unique information about molecular oxygen content inside the lungs. GASMAS may have a future role in optimising respiratory management of neonates in different clinical scenarios such as monitoring cardiorespiratory transition in the delivery room, assessing surfactant deficiency, and optimising endotracheal tube positioning. This article aims to summarise current evidence exploring GASMAS application in a neonate, discuss possible clinical benefits, and compare with other devices that are currently used in neonatal care.

**Impact:**

- This article presents a novel optical technique to measure lung oxygen concentrations that may have important clinical uses.
- This review summarises the current literature investigating the concept of optical lung oxygen measurement.
- Information from this review can guide researchers in future studies.

## Introduction

Medical applications of laser light technology are an ever-expanding area, enabling the improvement of non-invasive diagnostic and treatment techniques in different areas of medicine. In particular, a technique such as gas in scattering media absorption spectroscopy (GASMAS) has the potential to become a beneficial respiratory monitoring tool in clinical practice. Initially, GASMAS has been employed in different commercial areas (e.g., food,^1–4^ pharmaceutical industries^5–7^) helping to detect, measure and analyse gas trapped inside diffuse materials. More recent in vitro and in vivo studies evaluated the concept of non-invasive oxygen detection inside biological tissues like sinuses,^8–13^ middle ear,^14,15^ femoral bone^16^ and lungs. Different lung phantom models were used for GASMAS measurements, such as porcine lung samples,^17^ or produced by 3D printing nylon shells filled with tissue optical phantom material and air^18,19^ or structured sponge.^20^ An in vivo study has demonstrated GASMAS effectiveness in detecting pneumothorax and lung collapse in ventilated piglets.^21^ Following feasibility studies involving infants,^22,23^ GASMAS have been identified as a potential respiratory monitoring tool that might play a role in neonatology, managing infants in delivery room and intensive care units, where quantitative, non-invasive bedside monitoring of sick newborn infants is essential.

Respiratory illness is the most common indication for admission to the neonatal unit.^24^ Whilst up to 10% of term and late preterm infants born between 34 and 40 weeks gestational age can have respiratory problems,^24^ more than 80% of infants born before 28 weeks will require respiratory support.^25^ Reasons include delayed clearance of foetal lung fluid or aspiration of meconium for term babies and surfactant deficiency for premature neonates,^26,27^ compromising oxygen diffusion, absorption and subsequently resulting in decreased blood oxygen levels.

Neonatal respiratory status is currently monitored using continuous pulse oximetry (PO), intermittent blood sampling, and X-ray imaging. These methods cannot measure oxygen concentration or lung volume directly and carry some short and long-term risks.^28,29^ Optical oxygen concentration measurements present a clinical and diagnostic advantage allowing fast and non-invasive detection of suboptimal oxygen concentrations within the lung, subsequently allowing prevention of potential respiratory complications.

Currently, there is no commercially available GASMAS-based monitoring/diagnostic method that could be used in clinical practice to assess real-time regional ventilation and oxygen content at the bedside on a continuous basis.

This review article aims to summarise current evidence exploring neonatal oxygen monitoring of the lungs, discuss possible clinical applications of a novel GASMAS-based device and compare with other devices/techniques that are currently used in neonatal units.

## Gas in scattering media absorption spectroscopy

The most recurrent interactions between light and tissue are absorption and scattering (Supplementary 1 and 2). Characteristics of these interactions have been employed to develop optical technology called GASMAS. It is a technique, which measures the concentration of gas inside diffuse substances like biological tissue, relying on the difference between the absorption spectra of molecular gas and condensed matter (solids and liquids).^30^ A usual absorption band of biological tissue is 10^1^ nm broad versus 10^−4^ nm for gases. For a GASMAS measurement, a narrow-band diode laser at a specific wavelength is scanned across a characteristic gas absorption line to interrogate the material and gas pockets contained within.

The diffuse light emerging from this media carries the spectroscopic information about the gas that it passed through, which is recorded by a detector/sensor placed away from the light source. This information is used to calculate the gas concentration by means of the Beer–Lambert law, which states that the intensity of the emerging light (*I*), propagating through gas with concentration (*c*) and absorption cross-section (*ε*) along an optical path length (*l*), decays in an exponential way.

$$I = I_0e^{ - \varepsilon cl}$$

In cases where the optical path length (*l*) of the gas is unknown, a dual diode laser source is used to interrogate a reference gas present in the same cavity (e.g., water vapour). The optical path length can be assumed equal for both gases if their absorption bands are spectrally close.

**Fig. 1:**
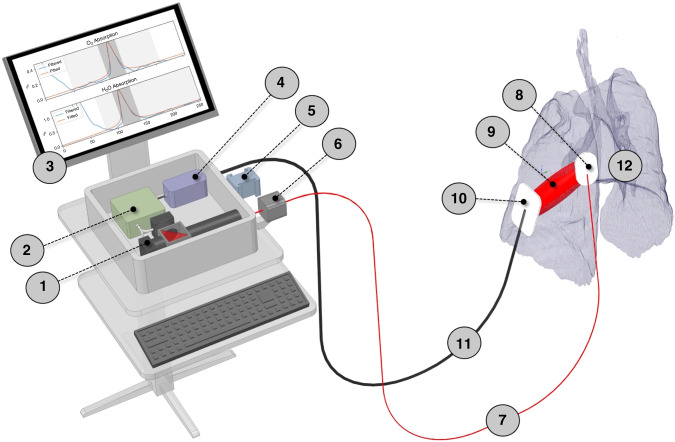
Schematic drawing of the GASMAS-based lung analyser system. 1 – Dual laser source; 2 – Data acquisition and laser control module; 3 – PC; 4 – Signal pre-amplifier; 5 – Reference measurement cube; 6 – Fibre interference eliminator; 7 – Optical fibre; 8 – Light source probe (emitter); 9 – Diffuse light passing through the lung tissue; 10 – Photosensitive detector probe; 11 – Electric cable; 12 – Neonatal lung.

**Fig. 2:**
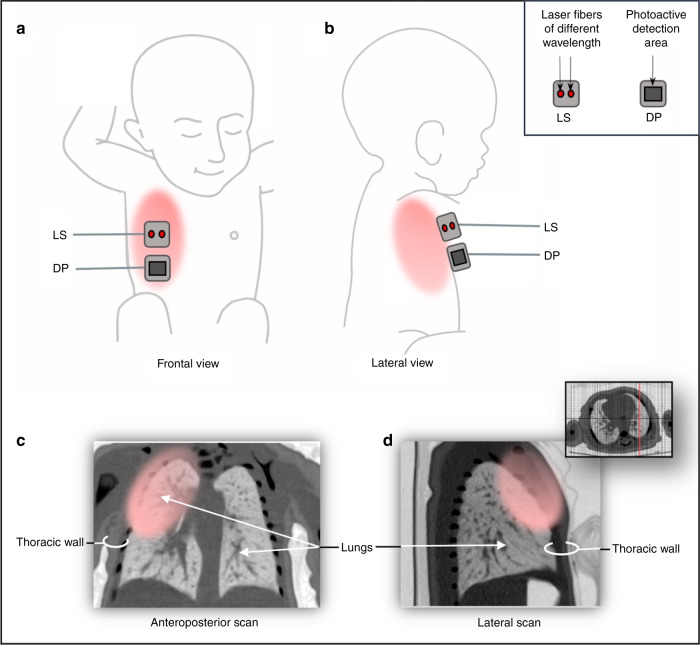
GASMAS clinical application for neonatal lung oxygen detection and monitoring. **a**, **b** Skin placement of the probes overlying lung area. LS - light source probe, DP - detector probe. **c, d** Illustration of light distribution using thoracic CT images of a full-term 4-week-old newborn as an example. Parental consent to use images obtained. Pink-coloured areas represent the approximate extent of near-infrared light illumination used for GASMAS. Given the difficulties in predicting photon migration, the areas of light illumination are not necessarily the same as the areas of light detection.

The novel clinical application of GASMAS technology is targeting the detection of molecular oxygen (O_2_) and water (H_2_O) vapour, common gases inside human body cavities, e.g., lung tissue. It uses a dual laser source with 760 and 935 nm to measure absorption signals from molecular O_2_ and H_2_O vapour, respectively. The concentration of H_2_O vapour in a cavity with 100% relative humidity and known central body temperature (like a neonate’s lung) can be calculated using the Arden Buck equation.^31,32^ Therefore, H_2_O vapour is used as a reference gas to estimate the optical path length and calculate the O_2_ concentration. Devices for the clinical application have the probe with integrated laser fibres attached to the skin as a light source and detector probe with the photosensitive area (Figs. 1 and 2).

## Oxygen measurement using GASMAS technology in clinical setting

### GASMAS applications in adult medicine

GASMAS have been applied in several feasibility studies to describe common adult diseases, like sinusitis, otitis media and osteonecrosis of femoral head. Sinusitis is an inflammatory condition resulting in a blockage of normal gas drainage and affecting the gas volume and content inside the sinus cavity due to mucosal swelling and production of secretions. The resulting blockage of normal gas drainage affects the gas volume inside the sinus cavity.

Persson et al. used two plastic plates simulating light transmission through sinuses. One plate acted as a human facial bone-muscle layer, and another plate like the base of the sinus cavity composed of mucous membranes and bone. By altering the thickness of the plates and the distance between them, it was possible to show that the O_2_ absorption signal was proportional to the distance between plates and O_2_ volume, thus allowing the detection of reduced gas content in cases of sinus pathology.^8^ Later Persson et al. demonstrated that GASMAS can be used to detect O_2_ within the maxillary and frontal sinuses in 11 volunteers. No O_2_ signal was detected where the volunteer was experiencing recurrent sinus issues. Also, the gas exchange process inside the sinus cavity has been investigated by flushing the sinuses with nitrogen, which cleared the cavity from O_2_. It has shown a ‘real-time’ O_2_ signal drop, indicating normal gas drainage due to non-obstructed connections between air pockets.^9^ Frontal sinus H_2_O signal repeatability analysis demonstrated that the recorded values remain consistent within one day or 6-day span with persistent right and left asymmetry.^13^ Further investigations have shown, that several gases (H_2_O vapour and O_2_) can be detected simultaneously.^11^

Thirty-one patients with suspected mastoid sinus pathology underwent laser spectroscopy measurements and the results were compared with subsequently performed computer tomography (CT) scans. Correlation was found between sinus volume based on CT scan grading and H_2_O vapour absorption imprints (*r*^2^ = 0.69, 47% of variance related). Weak reproducibility has been demonstrated for H_2_O vapour and molecular O_2_. Although, all cases with radiological signs of mastoid pathology were identified with laser spectroscopy.^12^

Middle ear phantoms have been used to prove the concept of GASMAS ability to detect changes consistent with otitis media diagnosis when air volume behind eardrum reduces due to increment secretions. O_2_ signal increases when flushing the phantom middle ear cavity with pure O_2_ and stays stable over time. It decreases gradually when opening the orifice of the cavity and reduces when flushed with nitrogen, demonstrating the relation between the signal and O_2_ concentration.^14,15^

Chen et al. demonstrated the role of GASMAS in the early detection of osteonecrosis of the femoral head. Femoral head decay is associated with forming of air-filled pores replacing normal vessels, which can be detected using GASMAS. Eighteen hip samples obtained after routine joint replacement surgery were investigated. Eleven of them were affected by osteonecrosis. Comparing water vapour signals from normal bones and affected, a significant difference (*P* < 0.05) was found illustrating in vitro detection efficacy. In vivo studies using arthroscopy with integrated laser is to follow by this research group.^16^

### Laser spectroscopy studies with infants

The first feasibility study performing lung measurements enrolled three healthy term-born babies. The study aimed to investigate the quality of signal detected by probes placed in different locations on the chest and abdomen. Most of the O_2_ and H_2_O signal received by the detector probe was weak. Poor signal quality was likely related to the thickness of the probed tissue. Because the measurements were performed on older babies (weighing above 4 kg) with a thicker subcutaneous layer, the light was likely to be scattered and attenuated before reaching the detector. However, one position gave a clear absorption signal for H_2_O vapour, with the laser source probe below the collar bone and detector just below the armpit. Few abdominal light source-detector positions were suitable to detect H_2_O vapour. The probe separation and absorption signal quality relationship was demonstrated by computer simulations.^33,34^

A subsequent study recruited 29 full-term healthy infants and obtained 390 lung optical measurements. The power of laser used for O_2_ detection has increased significantly (30 mW), compared to previous studies. A total of 60% of all measurements revealed good quality signals (SNR > 3), and O_2_ was detected at least once in each baby.^23^ Nevertheless, investigators suggested the need for technical improvements for the future development of a clinical device.

### GASMAS safety

The International Electrotechnical Commission and the American National Standards Institute have published a standard that outlines the safety of laser products. Laser light at the wavelengths and power level used for GASMAS falls under Class I category that is considered safe and presents no hazard to the eye or the skin under direct exposure. Laser light is coupled to the skin using a diffuser, which spreads the light over the larger surface area over the skin, reducing power density and maintaining skin and eye safety.

In previous animal and human models, the safety of the device hasn’t been reported. This is particularly important when applying long-term monitoring of vulnerable populations such as newborn, especially preterm babies that have immature skin. This needs to be factored into future study designs.

## Optical lung measurement versus other instrumental lung assessment techniques

Table 1 provides a comparative overview of the various technologies for lung evaluation available in the neonatal clinical setting.

**Table 1:** Comparison of GASMAS versus other oxygenation assessment and lung imaging methods used in clinical practice.

	Scanning time	Output	Ionising radiation	Bedside accessibility	Device cost^a^	Additional staff required	Skilled interpretation required	Motion sensitivity
GASMAS	Continuous	Numeric	No	Yes	Medium^b^	No	No	Unknown
Pulse Oximetry	Continuous	Numeric	No	Yes	Low	No	No	Yes
Chest X-ray	5 min	Images	Yes	Yes, if portable X-ray machine is available	High	Yes	Yes	Yes
Lung US	15–30 min	Images	No	Yes, if portable ultrasound machine is available	High	Yes	Yes	Yes
Lung CT	5 min	Images	Yes	Patient transfer required	Very high	Yes	Yes	Yes
Lung MRI	30–45 min	Images	No	Patient transfer required	Very high	Yes	Yes	Yes

*GASMAS* gas in scattering media absorption spectroscopy, *US* ultrasound, *CT* computer tomography, *MRI* magnetic resonance imaging.

^a^Grouped by cost for hospital-grade device as follows: very high >500,000 EU; high >100,000 EU; medium >10,000 EU; low >1000 EU.

^b^Estimated price of commercialised GASMAS device.

Chest X-ray is the most common method of evaluating the lung status of newborns. Radiologic appearance of different neonatal lung pathologies can be non-specific,^35^ and is caused by different constituents remaining inside the alveoli: hyaline membrane, foetal lung fluid or meconium. Gaining more information about the optical features of lung constituents may lead to the development of light-based technologies to enable more specific differentiation.

Lung ultrasound provides the possibility to image air-filled or collapsed parts of the lungs and is now more often utilised in neonatal units.^36–39^ While there are defined diagnostic criteria available for differentiation of various neonatal respiratory pathologies^40^ obtaining and interpreting these images requires training and a new skill set for the operators.

Lung CT greatly improves imaging of the pulmonary parenchymal and airway pathology and is considered as the gold standard for lung evaluation. Unfortunately, CT delivers significant concentrations of radiation compared to other radiological imaging techniques and has been found to be associated with increased risk for intracranial tumours.^41^ Furthermore, chest CT is resource and time intensive, requiring skilled personnel and effective patient immobilisation.^42^

While magnetic resonance imaging is generally considered safer than CT, its uses for neonatal lung evaluation are predominantly restricted to research settings only as spatial resolution quality is inferior to CT. Moreover, patient sedation is often necessary to avoid movement artefacts. Unlike the described radiological technologies, GASMAS would allow continuous monitoring at the cot side with comparatively easy interpretation of the results. In addition, GASMAS enables the detection of O_2_ at the molecular level and, with advancing technology, could also be used to analyse gas mixtures of O_2_, nitric oxide, or carbon dioxide.^43^

## Optical lung measurement versus other optical oxygen monitoring techniques: pulse oximetry and near-infrared spectroscopy

Neonatologists are familiar with other optical devices used daily in the neonatal units such as PO^44^ and near-infrared spectroscopy (NIRS). It is important to acknowledge the differences between PO, NIRS and GASMAS to understand how this new technology would add additional information about the overall O_2_ status of a neonate and what information is still missing to validate GASMAS. The main technical differences between these optical devices are demonstrated in Supplementary Fig. S3.

PO is used for continuous monitoring of the pulsatile haemoglobin bound O_2_ saturation (SpO_2_) signal that is a product of gas exchange at the alveolar level present in a peripheral vascular bed, while GASMAS targets free O_2_ molecules inside the gas pockets of the alveoli before they diffuse through the pulmonary capillaries and bind to haemoglobin. The light source of PO is emitting red and near-infrared light at the wavelengths specific to the absorption of deoxygenated (HHb) and oxygenated haemoglobin (HbO_2_). A single detector is positioned using diffuse transmittance geometry on the opposite side of the palm, wrist, feet, or ankle. Measurements obtained are dependent on pulsatile activity: light absorption changes with increasing blood volume present under the light emitter during systolic phase of cardiac cycle and reduces with diastole. As most of these rhythmic changes are associated with arterial vessel dilatation and constriction, PO is calculating arterial O_2_ saturation, based on red and infrared light absorption ratio. If pulse signal is lost (e.g., due to probe displacement or in cases of non-pulsatile or bradycardic conditions), measurement of SpO_2_ becomes unreliable.^45^

NIRS is used to monitor regional tissue oxygenation status, most commonly assessing neonatal brain tissue, by placing the probe on the infant’s forehead. It operates using the non-pulsatile, continuous wave (CW) signal within the same tissue optical window (Supplementary Fig. S2), penetrating brain tissue to a depth of 1–3 cm.^46^ Unlike PO, NIRS values are based predominantly on venous HbO_2_ saturation rather than arterial, because most of the haemoglobin is found within the venous compartment.^47^

NIRS measurements can be achieved using three different modes: CW, frequency domain (FD) or time domain (TD).^48,49^ CW-NIRS uses a single emitter delivering steady light and two or more detectors with a specific distance. The photodetector is sensing the light coming back from the tissues after interacting with HbO_2_ and HHb. Analysing the ratio between the intensity and frequency of diffusely reflected light it calculates the regional percentage of brain O_2_ saturation (RStO_2_).^46^

TD-NIRS relies on the ability to measure the photon distribution of time-of-flight in a diffuse media, following the emission of pulsed light with a duration of tens of picoseconds. Analytical algorithms provide values of HbO_2_, HHb, total amount of haemoglobin and RStO_2_, by estimating the absorption coefficient at two or more wavelengths.^50^

NIRS measurements incorporating FD use multiple emitter-detector pairs and estimate absolute values by evaluating phase shift and time-of-flight information.

GASMAS measurements are performed using two wavelength light sources specific to H_2_O vapour and molecular O_2_ absorption and a single detector. It is not targeting the absorption differences between HbO_2_ and HHb, like PO and NIRS. The calculation of O_2_ concentration using GASMAS is described in paragraph 2. While the distance between the emitter and detector as well as geometry (reflectance or transmittance) are well defined in PO and NIRS, there is a lack of studies confirming optimal probe placement for GASMAS measurements. Furthermore, optimal positioning on the chest is not well validated (Supplementary Table S1). Other factors that possibly would influence signal detection, like patient movement, cardiac contractility, breathing activity have not been investigated to date.

## Potential clinical applications of GASMAS in neonatal care

### Cardiorespiratory transition monitoring in the delivery room

Current clinical practice to maintain optimal oxygenation of a preterm baby in the delivery room is based on SpO_2_ and electrocardiogram derived heart rate values. Not reaching an SpO_2_ of 80% at 5 min postnatally was found to be associated with adverse outcomes, including intraventricular haemorrhage.^51^ It has been shown that despite using SpO_2_ and heart rate-driven O_2_ titration strategies, many infants spend a significant portion of time outside of the targeted O_2_ saturation range.^52,53^ Delayed availability of PO values,^54^ inaccurate values in bradycardic situations and lack of information about the actual efficacy of lung ventilation and aeration contribute to this finding. Lung recruitment is the crucial first step of neonatal transition and resuscitation. There is no current standard monitoring method of lung ventilation in the delivery room. Tidal volume monitoring is feasible, but challenging^55,56^ and can be inaccurate, especially in high mask leak situations. Non-invasive lung O_2_ monitoring using an optical device may provide ‘real-time’ information on lung ventilation, and subsequently optimise respiratory management during the dynamic situation soon after birth.

### Endotracheal tube placement

Endotracheal (ET) intubation is a high skill requiring procedure performed by clinicians in the delivery room and the neonatal intensive care unit. The success rate of correct ET tube placement on the first attempt is 24% by paediatric trainees, 52% by fellows, 64% by neonatologists.^57^ Chest radiography as well as clinical signs and exhaled carbon dioxide detector are commonly used to confirm the correct ET tube position. On the other hand, each monitoring modality has some limitations, for example, the end-tidal colorimeter may not change in colour if there is no cardiac output, but the ET tube is in the correct place; gastric secretions may cause the device to change colour, but the ET tube is in the oesophagus instead of trachea. While correct and prompt ET tube position confirmation is critical to avoid complications due to delayed effective ventilation, continuous non-invasive O_2_ monitoring could be beneficial. Optical monitoring of O_2_ content could reveal asymmetrical O_2_ distribution within a chest suggesting ET tube tip located too deep in one of the main bronchi or no O_2_ detected would prompt the clinician to consider a misplaced ET tube.

There are three techniques currently used to estimate the depth of ET tube insertion to ensure optimal midtracheal position. The first is based on the calculation of estimated distance from the lip to midtracheal location (in centimetres) using equation 6 + baby’s weight (kg). Second, guided by a vocal cord mark present on the tube. And third, palpation of the ET tube tip at the suprasternal notch. The proportion of correctly positioned ET tubes was found to be similar using either of these techniques (suprasternal palpation 47%, weight 38–44%, vocal cord guide 40%). In all, 82–87% incorrectly positioned ET tubes were too low.^58,59^ This demonstrates the need for a more efficient method to detect the position of ET tubes in neonatal care.

It has been demonstrated that by simulating atelectasis of one lung because of ET tube malposition, GASMAS technology has the potential to detect the difference of gas content (reduced on the side of atelectasis) with suddenly dropping O_2_ absorption value.^21^ Moreover, prompt technique could allow monitoring of ET position in cases when X-ray imaging is not available.

### Surfactant therapy

Surfactant therapy plays an essential role predominantly in the management of premature babies with RDS that is affecting approximately 80% of babies born at 28 weeks’ gestation.^60^ RDS causes diffuse alveolar collapse and compromises O_2_ delivery to the body. Multiple researchers are seeking to find an ideal standard of the best timing, dose, and methods of delivering surfactant treatment to maximise effect and minimise risks. GASMAS technology could help to detect early O_2_ level changes inside the lungs to determine optimal timing of surfactant administration. Moreover, different methods of delivering surfactant could be investigated by continuous real-time monitoring. A previously reported study has demonstrated that GASMAS technology can detect gas volume change.^13^ Furthermore, it is known that equally diffused spread of surfactant inside proximal airways is essential for the therapy to be effective.^61^ While distribution depends on multiple factors (instillation technique, speed, volume of preparation, ET tube position, ventilation strategy, gravity),^62^ continuous measurements of lung O_2_ would be the first available and informative method to monitor the surfactant administration procedure and potentially optimise the treatment algorithms.

### Patient positioning improving lung ventilation

Multiple adult and animal models have shown gravity-dependent lung inhomogeneity with gas exchange and ventilation/perfusion matching improving when treated in the prone position. In the presence of an abdominal distension, which is a common issue for neonates, the prone position has a positive effect on gas exchange as well.^63^ Infants that require assisted ventilation are regularly repositioned to improve lung gas exchange. Studies comparing different neonatal positions have shown that arterial O_2_ saturation increases if the ventilated baby is being nursed in a prone or prone alternant position (*P* < 0.001).^64,65^ While baby’s body position has an impact on cardiopulmonary function,^65^ it is still unclear when the position should be changed, how often and which positions should be chosen. More knowledge about the changes in lung inflation obtained from continuous optical monitoring is required to give us the answers to the above questions.

### Bronchopulmonary dysplasia diagnostics

Bronchopulmonary dysplasia (BPD) is a long-term adverse outcome of preterm delivery and often requires prolonged ventilation support and O_2_ therapy. Histopathological findings of BPD include alveolar simplification, dysmorphic capillaries, increased in vascular and airway smooth muscle cells, abnormal deposition of the extracellular components (e.g., elastin and collagen) and interstitial fluid accumulation.^66^ All these changes cause alveolar collapse (atelectasis) and compensatory overexpansion, subsequently impairing gas exchange and causing dependency on respiratory support and O_2_ therapy. BPD diagnosis has been evolving over time, initially based on histopathological findings, later relying on the duration of O_2_ supplementation, the amount of O_2_ required and most recently adding criteria of positive pressure ventilation support at ≥28 days post-natal age or at ≥36 weeks post-menstrual age.^67^ GASMAS with multisite measurements could be used to measure lung volume and O_2_ content to optimise diagnostic and management strategies for babies with BPD. To our knowledge, to date, no studies have reported the application of multiple light sources and detector probes and measuring signals simultaneously.

## Conclusions

GASMAS-based devices have potential clinical use in neonatology, especially in the assessment and management of various respiratory conditions. Currently, only a few studies have assessed its feasibility in the neonatal population. More findings are available from studies performed with phantoms and will add important information on GASMAS applicability and required improvements in the future. Compared with other devices currently used in clinical practice for lung function monitoring, GASMAS has the advantages of bedside availability, continuous monitoring option, and unique information about molecular oxygen content inside the lung. Future research should aim to validate GASMAS measurements to prove reliability and precision.

## Supplementary information

Supplementary Figure S3_with legend_for submission_DPI300

Supplementary 1 and 2_for submission

Supplementary Table 1_for submission
