# Supplementary material for: Gas in scattering media absorption spectroscopy as a potential tool in neonatal respiratory care
Source: Pediatr Res. 2022 May 23;92(5):1240–6. doi: 10.1038/s41390-022-02110-y (PMC9700509; doi:10.1038/s41390-022-02110-y)
Supplement: Supplementary file 1 — Supplementary Figure S3_with legend_for submission_DPI300 [file 41390_2022_2110_MOESM1_ESM.pdf]

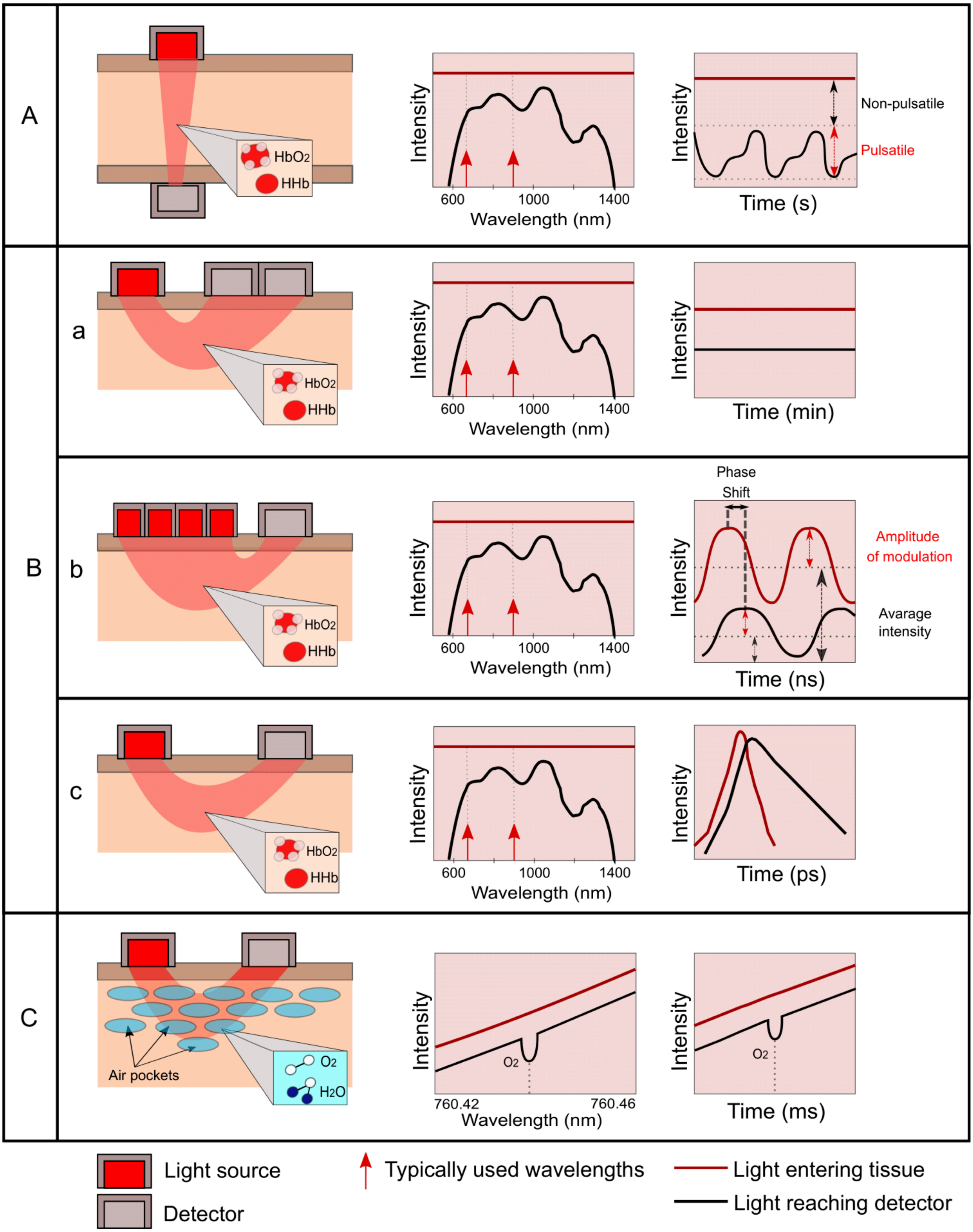

Supplemental Figure S3. Schematic comparison of technical characteristics of optical devices: A - Pulse Oximeter; B (a) - Continuous Wave NIRS; B (b) - Frequency Domain NIRS; B (c) – Time Domain NIRS; C - GASMAS. First column of each panel is visual depiction of probe (light emitter and detector) placement and light distribution through tissues. Second and third columns are graphical representation of changes of emitted light and detected light intensity depending on wavelength, and overtime measurements. Abbreviations: HbO_2 – oxygenated haemoglobin; HHb – non-oxygenated haemoglobin; O_2 – oxygen molecule; H_2O – water vapor molecule.
