# Supplementary material for: Gas in scattering media absorption spectroscopy as a potential tool in neonatal respiratory care
Source: Pediatr Res. 2022 May 23;92(5):1240–6. doi: 10.1038/s41390-022-02110-y (PMC9700509; doi:10.1038/s41390-022-02110-y)
Supplement: Supplementary file 2 — Supplementary 1 and 2_for submission [file 41390_2022_2110_MOESM2_ESM.pdf]

SUPPLEMENT 1. Light-tissue interactions: absorption and scattering

The tissue optical properties describe the interaction features between light and human body constituents such as water, lipids, proteins, carbohydrates, nucleic acids and minerals. The different size of the cells, their orientation and structure impact the possible interactions of tissue with the electromagnetic spectrum (light of different colors). The most recurrent interactions between light and tissue are *absorption* and *scattering*.

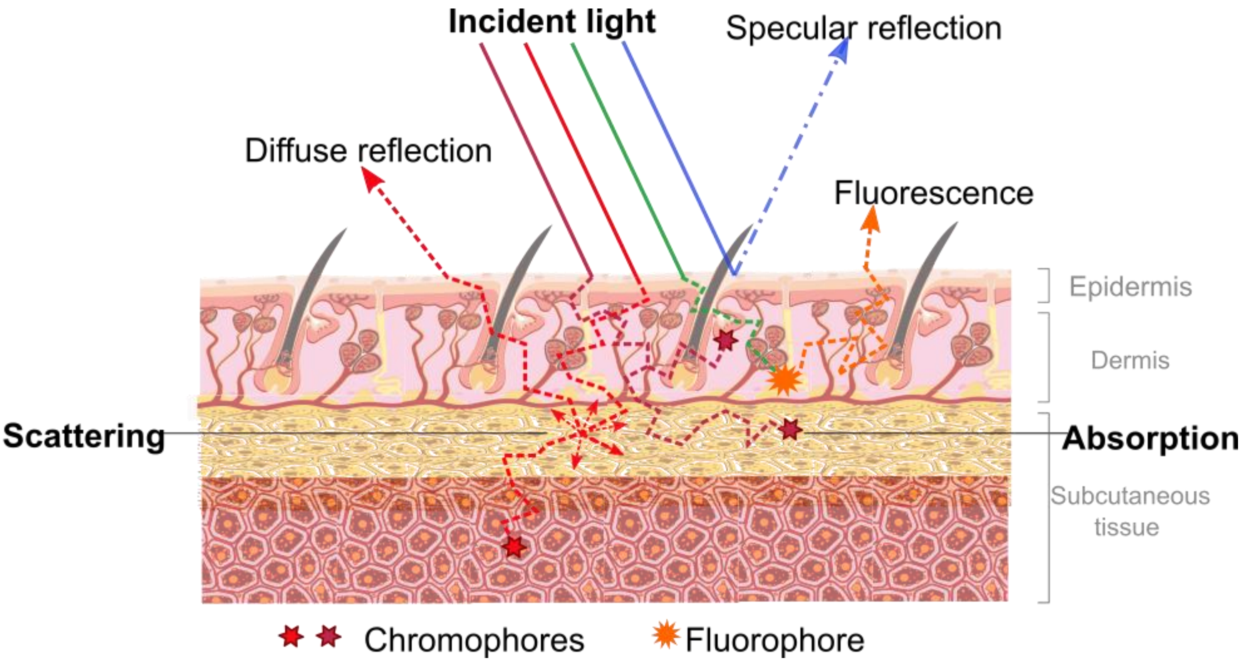

Supplemental Figure S1. Types of light and tissue

Absorption happens when the photon of incoming light, transfers energy to the tissue molecules, by means of photo-thermal, photo-mechanic or photo-chemical effect. Special parts of tissue molecules like chromophores and fluorophores are taking part in this process. Chromophores absorb UV or visible light and reflect specific wavelengths within the visible region, resulting in characteristic coloration of biological tissues (e.g., red for blood, yellowish for fat). Fluorophores, on the other hand, are emitting longer wavelength with lower energy light after exposure. This process is called fluorescence and gives glowing appearance of material.

After the contact with the tissue, the photon may be changing the direction of the flight. This effect is called scattering. Biological tissue is characterized as a highly scattering material, which results in diffuse reflectance and transmission that occurs after a number of scattering events, causing the light to escape the tissue onto the same or opposite surface to the light source (Supplemental Figure S1).

SUPPLEMENT 2. Light-tissue interaction: tissue optical window

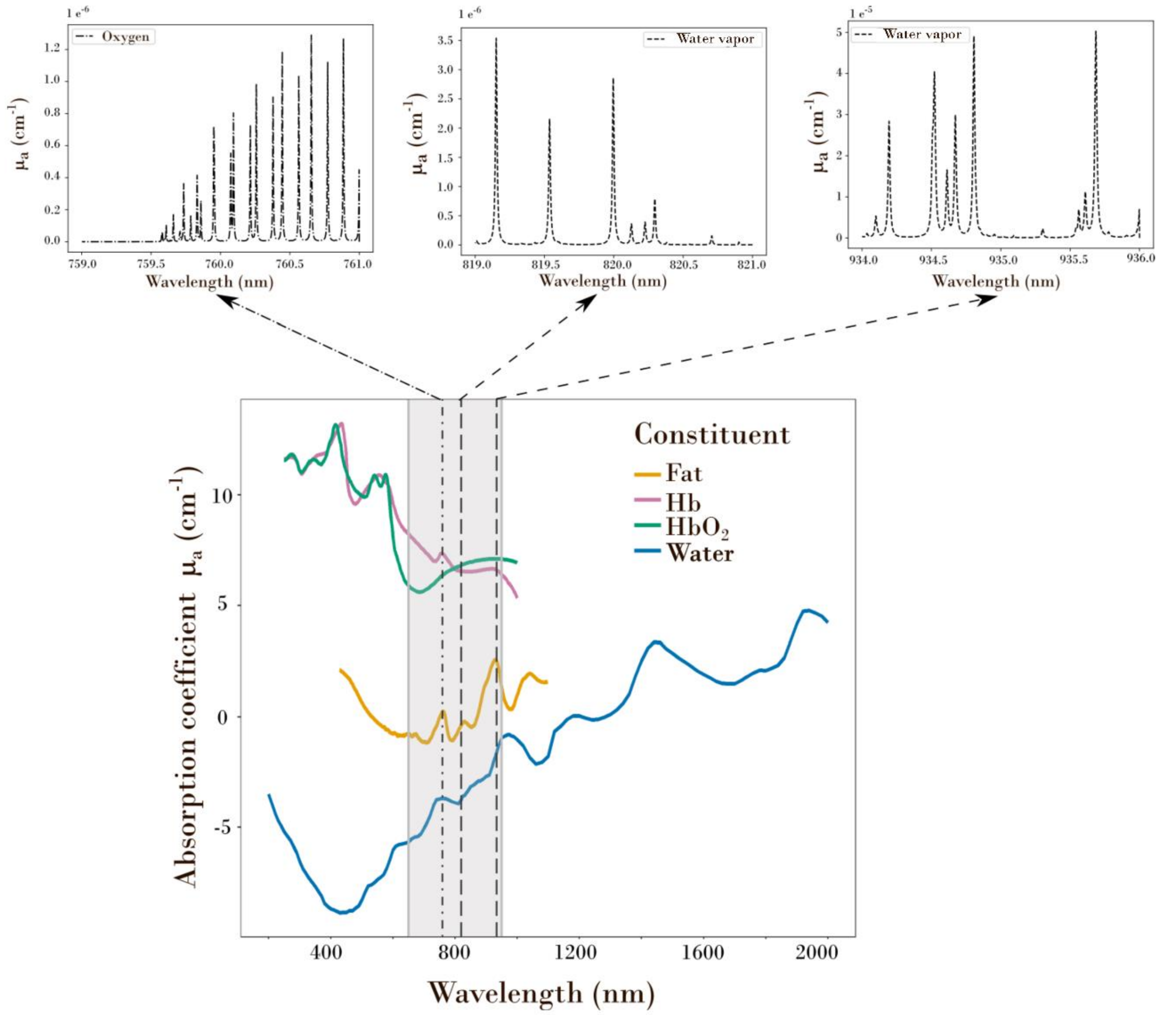

***Supplemental Figure S2**. **Absorption coefficient spectra from the main constituents in biological tissue.** The area between the vertical grey lines represents the tissue optical window. The dot-dashed line outlines molecular oxygen absorption region in gas phase at around 760 nm. The dashed lines representwater vapor absorption regions around 820 and 935 nm. Data reproduced from Prahl et al.[1] The figures on the top correspond to the absorption lines specific to water vapor and oxygen[2].*

However, absorption and scattering of photons causes attenuation of light passing through tissue. This significantly impacts how deeply light can penetrate and whether it can be measured on its return to the surface. Supplemental Figure 2 shows the absorption coefficient spectra for the main constituents of biological tissue. The region between grey lines, where light exhibits minimal absorption and optimal scattering, is defined as the first *tissue optical window*. As a result, light with wavelengths between 650-950 nm penetrates deeper in tissue because the overall absorption by fat, hemoglobin, oxyhemoglobin and water is relatively lower[3]. Consequently, laser light within this optical window can be used to interrogate biological tissues.

References:

1 Prahl, S. J. a. S. *Orgon Medical Laser Center.*, <https://omlc.org/spectra/index.html> (2018).

2 SpectraPlot.com: Integrated spectroscopic modeling of atomic and molecular gases. Available from: https://www.spectraplot.com/.

3 Boudoux, C. *Fundamentals of Biomedical Optics: From Light Interactions with Cells to Complex Imaging Systems* (Pollux, 2017).
